# Radiosynthesis, Biological Evaluation, and Preclinical Study of a ^68^Ga-Labeled Cyclic RGD Peptide as an Early Diagnostic Agent for Overexpressed *α*_v_*β*_3_ Integrin Receptors in Non-Small-Cell Lung Cancer

**DOI:** 10.1155/2020/8421657

**Published:** 2020-03-31

**Authors:** Nazanin Pirooznia, Khosrou Abdi, Davood Beiki, Farshad Emami, Seyed Shahriar Arab, Omid Sabzevari, Zahra Pakdin-Parizi, Parham Geramifar

**Affiliations:** ^1^Department of Radiopharmacy, Faculty of Pharmacy, Tehran University of Medical Sciences, Tehran, Iran; ^2^Drug Design and Development Research Center, Tehran University of Medical Sciences, Tehran, Iran; ^3^Research Center for Nuclear Medicine, Tehran University of Medical Sciences, Tehran, Iran; ^4^Nuclear Medicine and Molecular Imaging Department, Imam Reza International University, Razavi Hospital, Mashhad, Iran; ^5^Department of Biophysics, Faculty of Biological Sciences, Tarbiat Modares University, Tehran, Iran; ^6^Department of Toxicology and Pharmacology, Faculty of Pharmacy, Toxicology and Poisoning Research Centre, Tehran University of Medical Sciences, Tehran, Iran; ^7^Toxicology and Poisoning Research Centre, Tehran University of Medical Sciences, Tehran, Iran

## Abstract

The *α*_v_*β*_3_ integrin receptors have high expression on proliferating growing tumor cells of different origins including non-small-cell lung cancer. RGD-containing peptides target the extracellular domain of integrin receptors. This specific targeting makes these short sequences a suitable nominee for theranostic application. DOTA-E(cRGDfK)_2_ was radiolabeled with ^68^Ga efficiently. The *in vivo* and *in vitro* stability was examined in different buffer systems. Metabolic stability was assessed in mice urine. *In vitro* specific binding, cellular uptake, and internalization were determined. The tumor-targeting potential of [^68^Ga]Ga-DOTA-E(cRGDfK)_2_ in a lung cancer mouse model was studied. Besides, the very early diagnostic potential of the ^68^Ga-labeled RGD peptide was evaluated. The acquisition and reconstruction of the PET-CT image data were also carried out. Radiochemical and radionuclide purity for [^68^Ga]Ga-DOTA-E(cRGDfK)_2_ was >%98 and >%99, respectively. Radiotracer showed high *in vivo*, *in vitro*, and metabolic stability which was determined by ITLC. The dissociation constant (*K*_d_) of [^68^Ga]Ga-DOTA-E(cRGDfK)_2_ was 15.28 nM. On average, more than 95% of the radioactivity was specific binding (internalized + surface-bound) to A549 cells. Biodistribution data showed that radiolabeled peptides were accumulated significantly in A549 tumor and excreted rapidly by the renal system. Tumor uptake peaks were at 1-hour postinjection for [^68^Ga]Ga-DOTA-E(cRGDfK)_2_. The tumor was clearly visualized in all images. [^68^Ga]Ga-DOTA-E(cRGDfK)_2_ can be used as a peptide-based imaging agent allowing very early detection of different cancers overexpressing *α*_v_*β*_3_ integrin receptors and can be a potential candidate in clinical peptide-based imaging for lung cancer.

## 1. Introduction

Lung cancer is among the most frequently occurring and deadly cancers affecting both sexes [1]. It is also responsible for the largest number of deaths due to the poor prognosis of this type of neoplasm. The lung cancer death rate exceeds that of the main cancer types (colon, breast, and pancreas) with a 5-year survival of 17.8% [1, 2].

Lung cancers are divided into two major types of small-cell lung carcinoma (SCLC) and non-small-cell lung carcinoma (NSCLC). NSCLC accounts for 80% to 85%, and SCLC is responsible for 15% to 20% of lung cancers [3].

Membrane proteins are the characteristic feature of a cancer cell. The overexpressed membrane receptors have critical importance in cancer diagnosis and therapy. It is possible to select and design new molecules that can be attached specifically to these cell-surface proteins and detect/destroy cancer cells [4].

Molecular imaging targets used in the clinic must have high affinity for the overexpressed receptors and great tumor-to-organ ratio to deliver imaging probes to the tumor sites. Biological tumor-targeting agents include antibodies, proteins, peptides, and aptamers [5, 6].

Among the aforementioned tumor-targeting ligands, peptides benefit from high selectivity, high potency, fast blood clearance, and low toxicity, and nonimmunogenicity (excellent tolerability by patients), broad range of targets, better intratumoral diffusion owning to their lower molecular weight, and peptides are easy and quite inexpensive to synthesize and allow numerous conjugation possibilities for targeted delivery [7–9]. Therefore, targeting peptides can be an excellent option for the delivery of diagnostic and/or therapeutic agents and have favorable applications not only in neoplastic disease but also in other diseases [10].

Radiolabeled peptides have been extensively studied, and various peptide receptor imaging and targeted therapies have been performed. Peptides as radiotracer show appropriate characteristic such as metabolic stability and tolerance to changes during radiolabeling process [11, 12]. Different peptide receptors have been identified to overexpress on the cancer cell surface including the integrin receptor [13–15].

The integrin family regulates critical cellular functions in solid tumor progression and consequently made them an attractive target for cancer therapy/diagnosis. They are transmembrane *αβ* heterodimers, more than 20 different heterodimers, which are known in humans [16–20].

Two of the major integrins involved in NSCLC proliferation and metastasis are *α*_5_*β*_1_ and *α*_v_*β*_3_. As *α*_v_*β*_3_ integrin has high expression on tumor cells and *α*_v_ integrins contribute to angiogenesis and tumor progression, *α*_v_*β*_3_ integrin can be considered as an excellent target for early detection, treatment, and prognostic marker for lung cancer. The *α* and *β* subunits of *α*_v_*β*_3_ integrin have different structural domains, and the extracellular domains from *α* and *β* subunits provide the binding site for the ligand [21–24]. The tripeptide RGD sequence binds to the interface of the *β*-propeller domain from the *α* subunit and the *βα*-domain from the *β* subunit ([Supplementary-material supplementary-material-1]) [25, 26].

In the last decade, various radiolabeled RGD peptides targeting integrin *α*_v_*β*_3_ have been examined [27–36]. RGD peptides have the capacity to act as diagnostic probes for early detection of growing tumors. Between cyclic and linear RGD variants, cyclic structures are preferred because they have more stability, recognize higher receptor population, and have higher affinity for *α*_v_*β*_3_ integrin. Also, as the targeting biomolecule, they deliver radionuclide to the overexpressed receptors on tumor cells [25–36].

From early studies, various diagnostic and therapeutic radiopharmaceuticals have been developed. Radiometals are an important component of most radiopharmaceuticals because of their nuclear properties. Their nuclear emissions, energies, half-lives' importance, availability, and cost are determining factors in their application for regular medical use [37–39]. Peptides used for targeted molecular imaging and therapy are mostly radiolabeled with ^99m^Tc, ^68^Ga, ^177^Lu, and so on. ^68^Ga, one of the most suitable positron emitter radionuclide, has a half-life of 68 min and is a generator-produced radionuclide. ^68^Ga-labeled peptides can be used in diagnostic imaging using PET. The ^68^Ga decay mode results in superior imaging in positron emission tomography (PET) [30–43].

Here, we assessed the radiosynthesis, quality control, and *in vivo* and *in vitro* behavior of [^68^Ga]Ga-DOTA-E(cRGDfK)_2_. *In vitro* specific binding, cellular uptake, and internalization were determined. The tumor-targeting potential of [^68^Ga]Ga-DOTA-E(cRGDfK)_2_ in a NSCLC xenograft mouse model was studied. The ability of this radiolabeled peptide to act as a PET tumor imaging agent in NSCLC was investigated in mice bearing A549 lung tumors.

## 2. Materials and Methods

### 2.1. Materials

DOTA-E(cRGDfK)_2_ was purchased from FutureChem (Seol, Korea) ([Supplementary-material supplementary-material-1]). ^68^Ga was obtained from a ^68^Ge/^68^Ga Generator System (1.85 GBq, Pars Isotope Co., Karaj, Iran).

All chemicals were obtained from Aldrich (Germany) and used without further purification. Normal saline, sodium acetate, methanol, ammonium acetate, trifluoroacetic acid (TFA), acetonitrile, acetone, hydrochloric acid, and sodium acetate used for radiolabeling were of high purity. Milli-Q water (ultrapure water (Type 1), resistivity 18.2 MΩ·cm at 25°C) was obtained from a Direct Q system (Millipore) and used for the preparation of all aqueous solutions and buffers.

Cartridges, sterile collection vials, and all cold standards were purchased from ABX (Advanced Biochemical Compounds, Germany).

Radioactivity was determined by an ionization chamber (PTW CURIEMENTOR 4). Radio-TLC was performed using chromatography paper impregnated with silica-gel (ITLC-SG, Agilent Technologies, Santa Clara, California). Analysis was carried out with a TLC scanner (miniGita; Raytest, Straubenhardt, Germany). Radio-HPLC was performed using an Agilent 1260 reverse-phase HPLC system equipped with a NaI (Tl) radiodetector (Gabi, Raytest, Germany) and a PC interface running service pack 2 software (Raytest, Straubenhardt, Germany). pH was measured using a pH meter (Knick, 765 Laboratory pH Meter, Germany). NaI (Tl) gamma detector (Delshid, Tehran, Iran) was applied for radioactivity measurements during animal biodistribution studies.

Non-small-cell lung carcinoma (NSCLC) cell line (A549) and Swiss mouse embryo fibroblast (NIH-3T3) cells were obtained from Pasture Institute of Iran.

Animals' studies were approved by the Research committee of Tehran University of Medical Sciences. PET images were obtained using the Siemens Biograph6 True-Point (trueV) PET/CT scanner (Siemens AG, Erlangen, Germany).

### 2.2. Methods

#### 2.2.1. Molecular Docking

Sequences of various RGD-containing peptides were retrieved from the protein databank (http://www.rcsb.org/). These sequences were docked onto the crystal structure of integrin *α*_v_*β*_3_ (PDB code: 1L5G) using the HADDOCK webserver [44, 45]. cRGDfK docking was also performed using HADDOCK webserver. 30 docking runs were performed. Residues Asp150, Asp218 of chain A, and Arg216 of chain B were defined as the integrin *α*_v_*β*_3_ active site ([Supplementary-material supplementary-material-1]). Following the completion of docking runs, the top structure from each cluster was selected based on the lowest Haddock score for further analyzes. The best 10 structures out of 30 were used for MD simulation.

#### 2.2.2. Molecular Dynamics Simulations

To simulate the interaction of integrin-binding motifs with integrin *α*_v_*β*_3_, MD simulations were performed using the top solution from each of the four HADDOCK structures.

The MD simulations were carried out for 20 ns using GROMACS 5.0.7 software package with CHARM36 force field in a cubic 15 *∗* 15 *∗* 15 nm box filled with water molecules and Na counter-ions ([Supplementary-material supplementary-material-1]).

After energy minimization, the system was equilibrated, and 20 ns MD simulations were carried out at the NPT. In all simulations, to keep all hydrogen bonds rigid, the SHAKE algorithm was used.

Analyses of RMSD, radius of gyration, temperature, density, and pressure have been carried out to confirm the stability of each MD simulation.

After ensuring the stability of MD simulations, MM-PBSA analysis for each complex was carried out to calculate the total energy and estimate the affinity of binding.

#### 2.2.3. Radiolabeling of DOTA-E(cRGDfK)_2_ with ^68^Ga

^68^Ga was obtained from a ^68^Ge/^68^Ga-sterile generator. Labeling was performed using a modular synthesizer (GRP 4V) (Scintomics, Fürstenfeldbruck, Germany) with single-use cassettes. For radiolabelling, ^68^Ga[Ga^3+^] eluate was added to a mixture of 1.5 ml HEPES buffer (1.5 M in H_2_O, pH 4.5–5) and 70 *μ*g peptide. The reaction was incubated at 125.4°C for 5 min. The labeled molecules were purified in-line with the labeling module using the Sep-Pak C18 Plus light cartridge (Waters, Milford, MA, USA) and filtered through a Cathivex-GV 0.22 *μ*m filter (Merck Millipore) for sterilization ([Supplementary-material supplementary-material-1]).

#### 2.2.4. Quality Control

The radiochemical purity was determined via reverse-phase HPLC and ITLC. HPLC was performed on an Agilent 1260 system using a C18 column 150 × 3 mm, 3 *μ*m (Thermo Fisher Scientific, Dreieich, Germany), and for radiochemical purity determination, a NaI (Tl) radiodetector (Gabi, Raytest, Straubenhardt, Germany) was used. The mobile phase consisted of gradient mixtures of acetonitrile and 0.1% aqueous TFA with a flow rate of 0.5 ml/min.

ITLC was performed by chromatography paper impregnated with silica-gel (ITLC-SG, Agilent Technologies) using ammonium acetate (1 M) and methanol (1 : 1) as the mobile phase. The strips were analyzed using the thin-layer chromatography scanner (miniGita, Raytest, Germany).

#### 2.2.5. *n*-Octanol/Water Partition Coefficient

In a 5 ml Eppendorf tube, 100 *μ*l of the radiolabelled peptide, 0.99 ml of PBS, pH 7.4, and 1 ml *n*-octanol were mixed. After intense vortexing for 8 min, the two layers were separated by centrifugation. Then, 500 *μ*l aliquots of aqueous and organic layers were transferred to a new tube, radioactivity was measured in a well-type gamma counter (Delshid Gamma counter, Tehran, Iran), and partition coefficient (log *P*) values were calculated (*n* = 3).

#### 2.2.6. *In Vitro* Peptide Stability Studies

The stability of [^68^Ga]Ga-DOTA-E(cRGDfK)_2_ peptides was determined by incubating the compounds in 0.01 M phosphate buffered saline (PBS), pH 7.4, %5 human serum albumin, and 0.1 M sodium acetate, pH 5.5. All the test tubes were incubated at 37°C with mild shaking in Thermomixer and analyzed by ITLC at different time intervals. Results were expressed as percent radiochemical purity (%RCP) yield.

To determine the peptide stability at 4°C, the compounds were mixed with human serum albumin and incubated for different time points followed by ITLC analysis. Results were expressed as percent radiochemical purity (%RCP) yield.

#### 2.2.7. Protein Binding

The protein binding properties of [^68^Ga]Ga-DOTA-E(cRGDfK)_2_ in blood were investigated by protein precipitation. 1ml of the labeled complex and 3ml of human plasma were mixed and incubated for 1 hour at 37°C. Then, an equal volume of 10% trichloroacetic acid (TCA) was added, and samples were centrifuged at 3,000 rpm for 10 min to separate serum from cells. Precipitate was resuspended in 5% TCA and centrifuged at 3,000 rpm for 10 min. Precipitate and the supernatant fractions were analyzed for radioactivity in a gamma counter. Protein binding of [^68^Ga]Ga-DOTA-E(cRGDfK)_2_ was expressed as the fraction of radioactivity bound to the protein, in percentage of the total radioactivity.

#### 2.2.8. Metabolic Stability

1 *μ*g of [^68^Ga]Ga-DOTA-E(cRGDfK)_2_ was injected via the caudal vein of 3 *Mus musculus* Swiss male mice. Urine was collected 30, 60, and 90 min postinjection and analyzed directly by radio-TLC (ITLC, Agilent Technologies, Inc.). Obtained chromatograms were compared with the [^68^Ga]Ga-DOTA-E(cRGDfK)_2_ chromatogram. The purpose of this study is to assess whether the peptide was metabolized *in vivo* or excreted unchanged.

#### 2.2.9. Internalization, Surface, and Nonspecific Binding in Lung Cancer Cell Line

2 × 10^6^ human lung adenocarcinoma A549 cells were seeded in a 24-well plate (10^5^ per well). After 24 hours of incubation at 37°C in a cell culture incubator, cells were treated by 1 *∗* 10^5^ CPM of [^68^Ga]Ga-DOTA-E(cRGDfK)_2_ for 15, 30, 60, 120, and 240 min. Cellular uptake was terminated by removal of culture media and 2X quick washing step with the ice-cold serum-free medium to remove unspecific binding. The nonspecific binding was measured in a gamma counter.

Cells were incubated with 0.5 ml/well of acid wash buffer (50 mM glycine buffer, 100 mM NaCl, pH = 2.8) at room temperature to remove surface-bound radioactivity. This step determines the membrane-bound radioligand and internalized radioligand. Then, the cells were lysed using 1N NaOH at 37°C for 10 min and harvested. The two fractions were measured in a gamma counter. Each experiment was done in triplicate.

#### 2.2.10. Cellular Uptake

*In vitro* binding experiment of the radiolabeled peptide was performed with human lung adenocarcinoma A549 (as integrin *α*_v_*β*_3_-positive) and Swiss mouse embryo fibroblast (NIH-3T3) cells (as the negative control). Cells were grown in a flask to confluency, and 5 *∗* 10^5^ cells were seeded in a cell culture plate and incubated at 37°C for 24 hours. 40 nM of [^68^Ga]Ga-DOTA-E(cRGDfK)_2_ was added in triplicate and incubated at 37°C for 1 hour. The incubation was terminated by removing culture media and washing with the ice-cold serum-free medium. Then, cells were removed by brief treatment with trypsin, and EDTA and radioactivity in the cell suspension were measured.

For the determination of [^68^Ga]Ga-DOTA-E(cRGDfK)_2_ specific binding, A549 cells were pretreated with 500-fold of unlabeled DOTA-E(cRGDfK)_2_ 30 min before the addition of labeled peptides.

#### 2.2.11. Dissociation Constant (*K*_d_) and Receptor Expression Level (*B*_max_)

The binding affinity of [^68^Ga]Ga-DOTA-E(cRGDfK)_2_ was determined by the saturation radioligand binding assay. [^68^Ga]Ga-DOTA-E(cRGDfK)_2_ with high radiochemical purity was prepared. Human lung adenocarcinoma A549 was seeded in cell culture plates (1 *∗* 10^5^ cells/well) and incubated at 37°C. Cells were treated with increasing concentration of the radiolabeled peptide (10, 30, 60, 100, 150, 200, and 250 nM) and incubated at 37°C for 60 min. DOTA-E(cRGDfK)_2_ was used as blocking solution in 500X concentration of the highest concentration of the radiolabeled peptide. To eliminate free radioactivity, cells were washed with the ice-cold serum-free medium. Cells with bound radioactivity were harvested with trypsin-EDTA solution (at 37°C for 3 min) and counted with a gamma counter. *K*_d_ and *B*_max_ values were calculated by the nonlinear regression algorithm (GraphPad Prism version 8.2.1 (441) for windows).

#### 2.2.12. Biodistribution Study in Normal Mice

100 *μ*g of [^68^Ga]Ga-DOTA-E(cRGDfK)_2_ in 100 *μ*L saline was injected via the caudal vein of 3 groups of 3 *Mus musculus* Swiss male mice. At 30, 60, and 90 min postinjection, mice were euthanized using a lethal dose of ketamine/xylazine. Cardiac puncture was performed to collect blood in a preheparinised syringe, and organs were excised, washed, weighed, and the radioactivity of each organ was determined using a well-type gamma counter. The organ uptake was calculated as percent of the injected dose per gram of organ tissue (%ID/g).

#### 2.2.13. Cell Culture

Human lung adenocarcinoma A549 (NCBI No: C137) and Swiss mouse embryo fibroblast NIH-3T3 (NCBI No: C156) cells were acquired from Pasteur Institute of Iran. Cells were grown to confluence at 37°C with %5 CO_2_ and %85 humidity in DMEM/F12 and RMPI 1640 supplemented with %10 fetal bovine serum (Gibco) and 1% penicillin/streptomycin, respectively.

#### 2.2.14. Biodistribution Studies in Tumor-Bearing Mice

100 *μ*g of [^68^Ga]Ga-DOTA-E(cRGDfK)_2_ formulated in 100 *μ*L saline was injected via the tail vein of 3 groups of 3 BALB/c mice. At 30, 60, and 90 min postinjection, mice were euthanized using a lethal dose of ketamine/xylazine, and blood was collected from the heart using a preheparinised syringe. Tumor and normal organs were excised, washed, weighed, and the radioactivity of each organ was determined using a well-type gamma counter. The organ uptake was measured as percent of the injected dose per gram of organ tissue (%ID/g). A blocking experiment was performed in the presence of a 500-fold excess of unlabeled DOTA-E(cRGDfK)_2_ 30 min before [^68^Ga]Ga-DOTA-E(cRGDfK)_2_ injection.

#### 2.2.15. Animal Model and PET-CT Imaging

6–8 weeks female BALB/c mice (with body weights of 20–25 g) were purchased from Royan Insitute (Amol, Iran). Animals were housed in wire cages under controlled conditions of temperature at 25°C, relative humidity around 50%, and 12/12 h light/dark cycles with food, and water was given ad libitum. 2 × 10^6^ cell suspensions of human lung adenocarcinoma A549 in 100 *μ*l PBS were injected subcutaneously in the right dorsal flank of each mouse. Palpable tumor diameter was measured using digital calipers twice weekly. When tumor size reached a mean tumor volume of 50–100 mm^3^, animals received a tail vein injection of 740 kBq of [^68^Ga]Ga-DOTA-E(cRGDfK)_2_ in 100 *μ*l normal saline. After different postinjection intervals, mice were scanned on the PET/CT scanner (Siemens Medical Systems). Anaesthetized (ketamine/xylazine) animals were placed in a prone position. 20 min PET (axial FOV 148 mm) scans were carried out followed by a CT scan (spatial resolution 1.25 mm, 80 kV, and 30 mAs). Scans were reconstructed using a filtered back projection algorithm. The reconstructed PET images were fused with CT images.

#### 2.2.16. Statistical Analysis

All values were expressed as mean ± standard deviation (SD) with statistical significance analyzed using one-way analysis of variance or *t*-test using a GraphPad Prism computer fitting program. Probability (*P*) values <0.05 were considered statistically significant.

## 3. Results

### 3.1. Molecular Docking

Various selected sequences for docking are summarized in [Supplementary-material supplementary-material-1]. Ten sequences with the lowest haddock score were used for molecular dynamics simulations.

### 3.2. Molecular Dynamics Simulations

#### 3.2.1. Root-Mean-Square Deviation (RMSD)

Root-mean-square deviation of the C*α* atoms relative to the starting structure was assessed to confirm the stability of MD stimulations. Results show an increase in RMSD during the first nanoseconds and become fixed and stable subsequently which guarantee the stability of each MD simulations. These data confirm that each structure has reached their final conformation ([Supplementary-material supplementary-material-1]).

#### 3.2.2. Radius of Gyration

Radius of gyration (Rg) correlates to the compactness of the structures, and if a protein is stably folded, it will likely maintain a relatively steady value. Radius of gyration in each MD simulation alters over time which is due to conformational changes to create a more compact/tense structure. The radius of gyration becomes relatively fixed and stable in the last 2 nanoseconds ([Supplementary-material supplementary-material-1]).

#### 3.2.3. Temperature and Density

The temperatures and densities ultimately became fixed and stable, representing further proof of simulation stability (Figures [Supplementary-material supplementary-material-1] and [Supplementary-material supplementary-material-1]).

#### 3.2.4. MM-PBSA Analysis

MM-PBSA analysis after MD simulations in GROMACS was performed to estimate the affinity of binding. The results are summarized in [Supplementary-material supplementary-material-1].

As the E(cRGDfK)_2_ sequence in the complex with *α*_v_*β*_3_ integrin has the lowest total energy of −2308.72 kJ/mole (the highest binding affinity to the receptor), this sequence has been selected for synthesis and further investigation.

### 3.3. Synthesis and Chemical Characterization

Nonradioactive DOTA-E(cRGDfK)_2_ ([Supplementary-material supplementary-material-1]) was synthesized by FuturChem (Korea) with HPLC purity of >99%.

### 3.4. Radiolabelling of DOTA-E(cRGDfK)_2_ with Gallium-68

**Figure 1:**
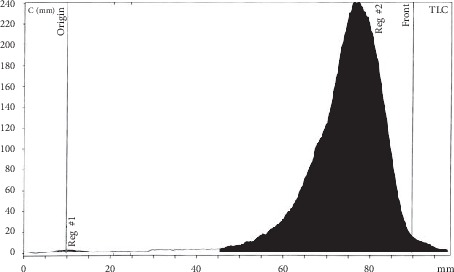
ITLC analysis of [^68^Ga]Ga-DOTA-E(cRGDfK)_2_. The radiochemical purity determined by radio-TLC was 99.7%.

**Figure 2:**
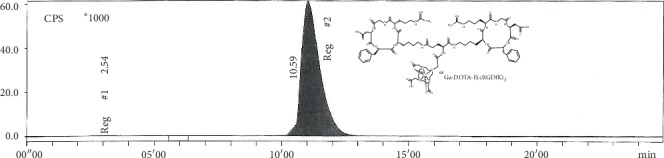
HPLC profile of [^68^Ga]Ga-DOTA-E(cRGDfK)_2_. The peaks at 2.54 and 10.59 min are related to free ^68^Ga and [^68^Ga]Ga-DOTA-E(cRGDfK)_2_, respectively.

[^68^Ga]Ga-DOTA-E(cRGDfK)_2_ was prepared in a high yield (%>98). The radiochemical purity determined by radio-TLC was >99.5% (Figure 1). HPLC profile of [^68^Ga]Ga-DOTA-E(cRGDfK)_2_ and the peaks at 2.54 and 10.59 min are related to free ^68^Ga and [^68^Ga]Ga-DOTA-E(cRGDfK)_2_, respectively (Figure 2).

### 3.5. *n*-Octanol/Water Partition Coefficient

The log *P* octanol/water values for [^68^Ga]Ga-DOTA-E(cRGDfK)_2_ were −3.968 ± 0.13. Results are presented as mean ± SD (*n* = 3).

### 3.6. *In Vitro* Peptide Stability Studies

**Figure 3:**
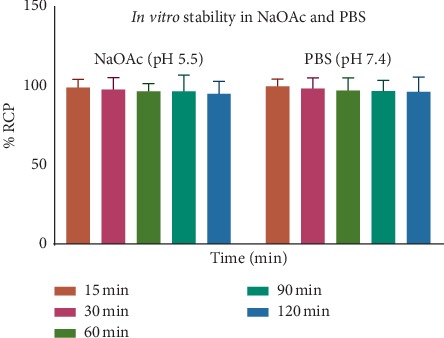
*In vitro* stability of [^68^Ga]Ga-DOTA-E(cRGDfK)_2_ in PBS and sodium acetate (NaOAc).

**Figure 4:**
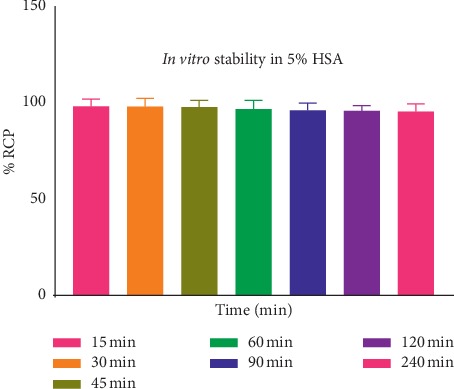
*In vitro* stability of [^68^Ga]Ga-DOTA-E(cRGDfK)_2_ in 5% human serum albumin (HSA) after storing at 4°C.

The stability of [^68^Ga]Ga-DOTA-E(cRGDfK)_2_ was determined at different time intervals by ITLC, as described above. The radiolabeled complex remained stable at 37°C, for up to 120 min for [^68^Ga]Ga-DOTA-E(cRGDfK)_2_ (Figure 3) postincubation. Stability studies in human serum albumin showed that >95% of [^68^Ga]Ga-DOTA-E(cRGDfK)_2_ remained intact up to 2 h postlabelling (Figure 4). Results show high stability and the suitability for *in vivo* experiments.

### 3.7. Protein Binding

The *in vitro* protein binding of [^68^Ga]Ga-DOTA-E(cRGDfK)_2_ was assessed in heparinized fresh human plasma and was found to be 2.88%.

### 3.8. Metabolic Stability

**Figure 5:**
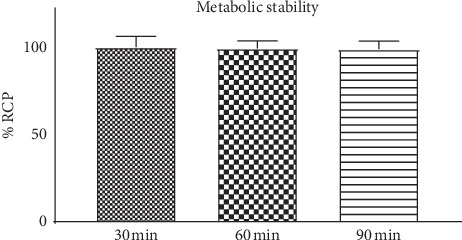
Metabolic stability of ^68^Ga-DOTA-E(cRGDfK)_2_.

The ITLC radiochromatograms of the [^68^Ga]Ga-DOTA-E(cRGDfK)_2_ peptide compared to appropriate urine samples showed good *in vivo* stability (Figure 5). The shape of the original peptide and urine sample chromatographic peaks is almost similar. The radiochemical purity showed >92% within 90 min postinjection. The percentage of radiochemical impurities is practically negligible.

### 3.9. Internalization, Surface, and Nonspecific Binding in Lung Cancer Cell Line

**Figure 6:**
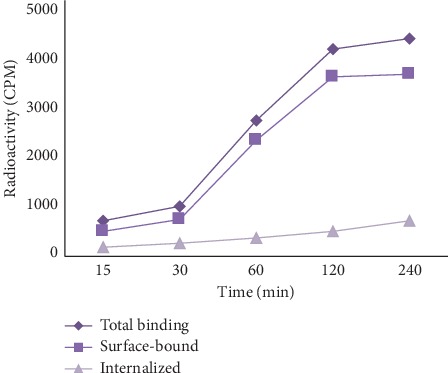
The total binding, surface-bound, and internalized radioactivity of [^68^Ga]Ga-DOTA-E(cRGDfK)_2_ at different time points.

For cell-binding assay, A549 cells (human adenocarcinoma cell line) were used. The human lung A549 cell that overexpressed *α*_v_*β*_3_ receptors may potentially serve as an important model for lung cancer. As shown in Figure 6, [^68^Ga]Ga-DOTA-E(cRGDfK)_2_ recognizes specifically *α*_v_*β*_3_ receptors and has high specific binding (internalized + surface-bound). Approximately, 16.5% of [^68^Ga]Ga-DOTA-E(cRGDfK)_2_ was internalized, and 83.5% of binding was surface-bound after 2 h incubation. The nonspecific binding was approximately 35%.

### 3.10. Cellular Uptake

**Figure 7:**
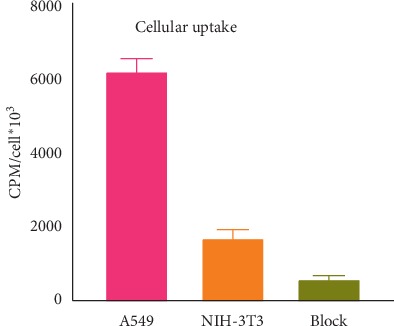
Cell uptake studies using human lung cancer A449 (*α*_v_*β*_3_-positive) and NIH-3T3 (*α*_v_*β*_3_-negative). Blocking studies using a 500-fold excess of unlabeled peptides confirmed receptor-specific uptake.

Compared to the A549 cell line, the binding of [^68^Ga]Ga-DOTA-E(cRGDfK)_2_ to NIH-3T3 (normal cell line) was low. Data from the competitive assay using 500-fold excess of the unlabeled peptide showed a very significant reduction in binding of [^68^Ga]Ga-DOTA-E(cRGDfK)_2_ to the A549 cell line which confirms the specific binding through the peptide (Figure 7).

### 3.11. Dissociation Constant (*K*_d_) and Receptor Expression Level (*B*_max_)

**Figure 8:**
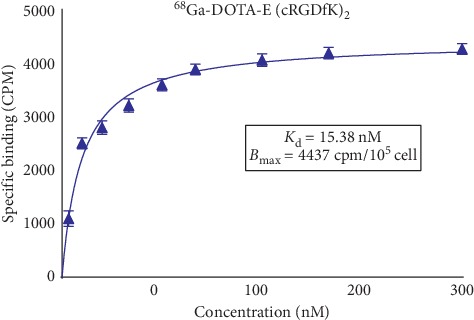
[^68^Ga]Ga-DOTA-E(cRGDfK)_2_ saturation binding assay curve.

Dissociation constant (*K*_d_) and receptor expression level (*B*_max_) of [^68^Ga]Ga-DOTA-E(cRGDfK)_2_ were determined on A549 cells (human adenocarcinoma cell line). *K*_d_ and *B*_max_ values are shown in Figure 8.

### 3.12. Biodistribution Study in Normal Mice

**Figure 9:**
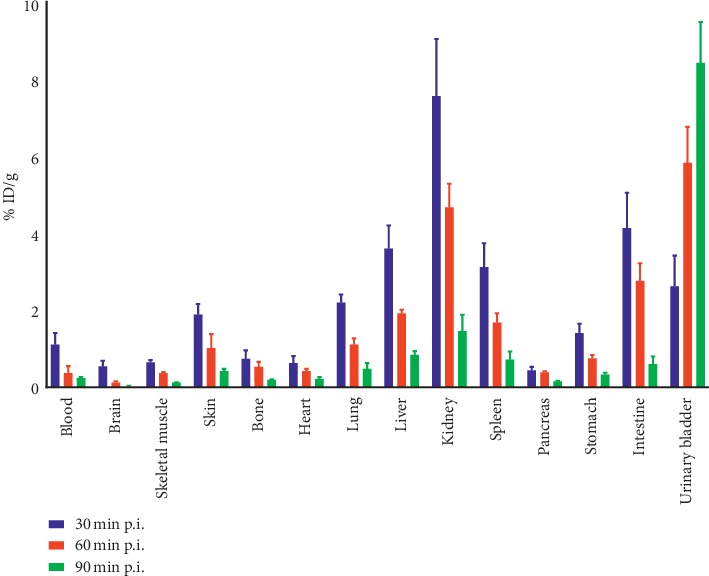
*In vivo* biodistribution of [^68^Ga]Ga-DOTA-E(cRGDfK)_2_ in normal mice. Data were calculated as a percentage of the injected dose per gram of organs (%ID/g) at 30, 60, and 90 min postinjection. Values are mean ± standard deviation (*n* = 3 per group).

Over time after injection, the radioactivity in tissues and organs decreased gradually (Figure 9). A quick reduction in the blood radioactivity level was seen with time. Significant uptake of [^68^Ga]Ga-DOTA-E(cRGDfK)_2_ was observed in kidneys, most likely due to excretion through the renal system.

### 3.13. Biodistribution Study in Tumor-Bearing Mice

**Figure 10:**
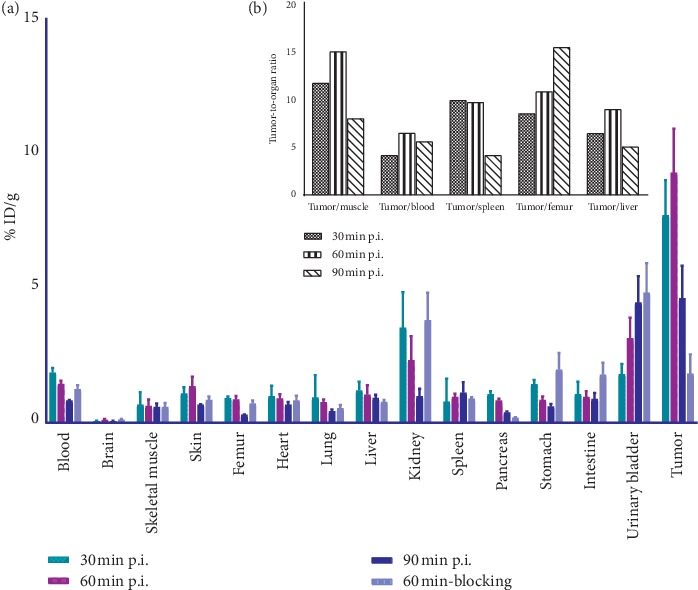
Biodistribution of [^68^Ga]Ga-DOTA-E(cRGDfK)_2_ in percentage of ID/g of organs at 30, 60, and 90 min postinjection in *α*_v_*β*_3_-positive tumor-bearing mice. Values are mean ± standard deviation (*n* = 3 per group) (a). Tumor-to-organ ratio at 30, 60, and 90 min postinjection (b).

The *in vivo* biodistribution of [^68^Ga]Ga-DOTA-E(cRGDfK)_2_ in A549 tumor-bearing mice is represented in Figure 10. As the tracer is highly hydrophilic, the clearance was fast and via the renal system. The radiolabeled peptide was accumulated in the tumor over time postinjection. In the blocking study, the coinjection of 500-fold excess of DOTA-E(cRGDfK)_2_ reduces the tumor uptake significantly.

### 3.14. Animal Model and PET Imaging

**Figure 11:**
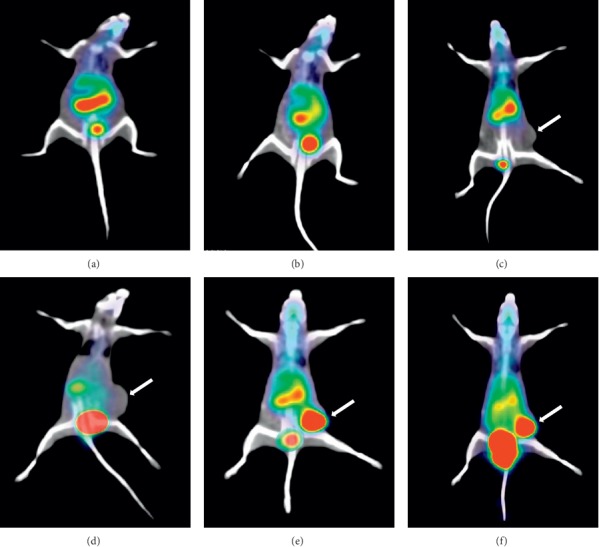
PET imaging of [^68^Ga]Ga-DOTA-E(cRGDfK)_2_ in normal mice at (a) 1 hour and (b) 2 hour after IV injection, 1 h (c) and 2 h (d) blocking using DOTA-E(cRGDfK)_2_, and PET images of tumor-bearing BALB/c mice at (e) 1 h and (f) 2 h after IV injection of [^68^Ga]Ga-DOTA-E(cRGDfK)_2_. White arrows are the indication of the tumor position.

All acquisitions using [^68^Ga]Ga-DOTA-E(cRGDfK)_2_ were carried out in A549 tumor-bearing BALB/c mice. Fused PET/CT scans are shown in Figure 11. PET images showed a high uptake of [^68^Ga]Ga-DOTA-E(cRGDfK)_2_ in the tumor, bladder, and kidneys in normal mice and those bearing A549 tumor cells (which is in accordance with the biodistribution data). The tumor is distinguishable on all PET/CT scans. High uptake of the radiopeptide in the kidney and bladder reflects the metabolic excretion of [^68^Ga]Ga-DOTA-E(cRGDfK)_2_ through the renal system in mice.

## 4. Discussion

Radiolabeled peptides have shown great promise for cancer therapy [12–15]. Peptides as radiotracer show appropriate characteristic such as metabolic stability and tolerance to changes during radiolabeling process [15]. Various receptors overexpressed on the cancer cell surface [4]. Integrin *α*_v_*β*_3_ has low expression on normal endothelial cells, but it is overexpressed on tumor cells. The integrin family regulates critical cellular functions in solid tumor progression, and consequently, they can serve as an attractive target for cancer therapy/diagnosis.

In the last decade, various radiolabeled RGD peptides targeting integrin *α*_v_*β*_3_ have been examined [27–36]. Between cyclic and linear RGD variants, cyclic structures are preferred due to their good stability. Also, as the targeting biomolecule, they carry radionuclide to the integrin *α*_v_*β*_3_ overexpressed on tumor cells [25–36].

Therefore, in recent years, many studies have focused on describing novel radiolabeled RGD peptides for molecular-based imaging [21–36].

In this work, the best RGD-containing peptide was selected. Sequences of various RGD-containing peptides were retrieved from the protein databank (http://www.rcsb.org/). These sequences were docked onto the crystal structure of integrin *α*_v_*β*_3_ (PDB code: 1L5G) using the HADDOCK webserver. As a result, 30 docking runs were performed. Residues Asp150, Asp218 of chain A, and Arg216 of chain B were specified as the active parts of integrin *α*_v_*β*_3_. Upon completion of the docking runs, the top solution from each generated cluster was analyzed. The selected structure cluster for the analysis was based on the lowest Haddock score without any restraint violations. The best 10 structures and the cRGDfK-integrin complex were used for MD simulation. MM-PBSA analysis shows that E(cRGDfK)_2_ has a total energy of −2308.72 kJ/mole which is the lowest total energy (highest binding affinity) among all complexes and justifies the reason for selecting this cyclic sequence for further investigation.

In the present study, we selected ^68^Ga for DOTA-E(cRGDfK)_2_ labeling due to its favorable physicochemical characteristics for imaging. ^68^Ga, a positron emitter radionuclide, has a suitable half-life of 68 min and is a generator-produced radionuclide. ^68^Ga-labeled peptides can be used by diagnostic imaging using PET. The ^68^Ga decay mode results in superior imaging in positron emission tomography (PET).

We synthesized [^68^Ga]Ga-DOTA-E(cRGDfK)_2_ in high radiochemical and radionuclide purity. The log *P* value was −3.968 ± 0.13 for [^68^Ga]Ga-DOTA-E(cRGDfK)_2_ which shows the highly hydrophilic property of radiolabeled E(cRGDfK)_2_ and mainly renal route of excretion.

[^68^Ga]Ga-DOTA-E(cRGDfK)_2_ showed very good *in vitro*, *in vivo*, and metabolic stability. The plasma protein binding of [^68^Ga]Ga-DOTA-E(cRGDfK)_2_ was low, <5%. The cellular uptake experiment of the radiolabeled peptide demonstrates very high uptake in A549 (as integrin *α*_v_*β*_3_-positive) in comparison to NIH-3T3 normal cell specific uptake which was confirmed by coinjection of the cold ligand.

^68^Ga-DOTA-E(cRGDfK)_2_ recognizes specifically *α*_v_*β*_3_ receptors demonstrating high specific binding characteristics (internalized + surface-bound). Approximately, 16.5% of the [^68^Ga]Ga-DOTA-E(cRGDfK)_2_ was internalized, and 83.5% of binding was surface-bound after 2 h incubation. The nonspecific binding was approximately 35%. The high specific binding is desirable for the application of [^68^Ga]Ga-DOTA-E(cRGDfK)_2_ in imaging and assessment of response to treatment.

Dissociation constant of [^68^Ga]Ga-DOTA-E(cRGDfK)_2_ was 15.38 ± 3.42 which demonstrates that only a low concentration of the ligand is required to occupy the receptors, indicative of high binding affinity. Based on previous studies, multimeric cyclic RGD has higher (20–100 more) *α*_v_*β*_3_ binding affinity than its monomeric alternatives [46]. In a study, *K*_d_ value for cRGDfK (as reference) was reported to be 1.3  × 10^−6^ M [47]. Many other works reported higher dissociation constant or lower affinity [48, 49]. Therefore, all the aforementioned data confirm the high binding affinity of [^68^Ga]Ga-DOTA-E(cRGDfK)_2_ to lung cancer tumor cells.

*In vivo* biodistribution results in A549 tumor-bearing BALB/c mice demonstrated significantly high tumor accumulation with low radioactivity retention in other organs, although moderate uptake of radioactivity was observed in the kidney and intestine.

PET/CT imaging of [^68^Ga]Ga-DOTA-E(cRGDfK)_2_ showed very high tumor uptake.

In conclusion, this study provides the first evaluation of the potential of [^68^Ga]Ga-DOTA-E(cRGDfK)_2_ for non-small-cell lung cancer imaging. Single dose of [^68^Ga]Ga-DOTA-E(cRGDfK)_2_ would be a promising diagnostic biological-based drug for cancer imaging, tumor treatment response monitoring, and follow-up imaging. Therefore, [^68^Ga]Ga-DOTA-E(cRGDfK)_2_ can serve as a great radiotracer for accurate and early detection of lung lesions.

## Data Availability

The data used to support the findings of this study are available from the corresponding author upon request.

## Supplementary Materials

Supplementary Materials Figure S1. Schematic representation for the extracellular part of integrin *α*_v_*β*_3_. RGD ligand binding site is the interface between *β*A-domain of the *β*_3_ subunit and *β*-propeller of the *α*_v_-subunit. Figure S2. The chemical structure of DOTA-E(cRGDfK)_2_ based on the amino acid sequence. Figure S3. Model for the interaction of cRGDfK with the RGD-binding site of *α*_v_*β*_3_. Figure S4. Structure of the integrin *α*_v_*β*_3_-RGD complex in the simulation 15 *∗* 15 *∗* 15 nm box. Red dots and blue spheres represent water molecules and ions, respectively. Figure S5. Schematic structure of ^68^Ga-DOTA-E(cRGDfK)_2_. Figure S6. RMSD values (C*α*-C*α*) during the MD production phase relative to the starting structure during 20 ns total simulation time. Figure S7. Radius of gyration as a function of time with respect to the starting structures during MD simulations. Table S1. Selected sequences for docking, number of amino acids, and HADDOCK score. Figure S8. Plots of the system temperature during simulations of the sequences. Figure S9. Plots of the system density during MD simulations. Table S2. Amino acid sequences, number of amino acids, and average total energy of the selected sequences.
