# Supplementary material for: Radiosynthesis, Biological Evaluation, and Preclinical Study of a 68Ga-Labeled Cyclic RGD Peptide as an Early Diagnostic Agent for Overexpressed αvβ3 Integrin Receptors in Non-Small-Cell Lung Cancer
Source: Contrast Media Mol Imaging. 2020 Mar 31;2020:8421657. doi: 10.1155/2020/8421657 (PMC7153000; doi:10.1155/2020/8421657)
Supplement: Supplementary Materials — Figure S1. Schematic representation for the extracellular part of integrin αvβ3. RGD ligand binding site is the interface between βA-domain of the β3 subunit and β-propeller of the αv-subunit. Figure S2. The chemical structure of DOTA-E(cRGDfK)2 based on the amino acid sequence. Figure S3. Model for the interaction of cRGDfK with the RGD-binding site of αvβ3. Figure S4. Structure of the integrin αvβ3-RGD complex in the simulation 15 ∗ 15 ∗ 15 nm box. Red dots and blue spheres represent water molecules and ions, respectively. Figure S5. Schematic structure of 68Ga-DOTA-E(cRGDfK)2. Figure S6. RMSD values (Cα-Cα) during the MD production phase relative to the starting structure during 20 ns total simulation time. Figure S7. Radius of gyration as a function of time with respect to the starting structures during MD simulations. Table S1. Selected sequences for docking, number of amino acids, and HADDOCK score. Figure S8. Plots of the system temperature during simulations of the sequences. Figure S9. Plots of the system density during MD simulations. Table S2. Amino acid sequences, number of amino acids, and average total energy of the selected sequences. [file 8421657.f1.pdf]

Electronic Supplementary Material

Radiosynthesis, biological evaluation and preclinical study of a ^{68}Ga-labeled cyclic RGD peptide as an early diagnostic agent for overexpressed $\alpha_v\beta_3$ integrin receptors in non-small cell lung cancer

Nazanin Pirooznia[1], Khosrou Abdi[1,2*], Davood Beiki[3], Farshad Emami[4], Seyed Shahriar Arab[5], Omid Sabzevari[6, 7], Zahra Pakdin-Parizi[4], Parham Geramifar[3]

[1]Department of Radiopharmacy, Faculty of Pharmacy, Tehran University of Medical Sciences, Tehran, Iran
[2]Drug Design and Development Research Center, Tehran University of Medical Sciences, Tehran, Iran
[3]Research Center for Nuclear Medicine, Tehran University of Medical Sciences, Tehran, Iran
[4]Nuclear Medicine and Molecular Imaging Department, Imam Reza International University, Razavi Hospital, Mashhad, Iran
[5]Department of Biophysics, Faculty of Biological Sciences, Tarbiat Modares University, Tehran, Iran
[6]Department of Toxicology and Pharmacology, Faculty of Pharmacy, Toxicology and Poisoning Research Centre, Tehran University of Medical Sciences, Tehran, Iran
[7]Toxicology and Poisoning Research Centre, Tehran University of Medical Sciences, Tehran, Iran

Corresponding author:
Khosrou Abdi, PhD, Department of Radiopharmacy, Faculty of Pharmacy, Tehran University of Medical Sciences, 16 Azar Street, Enghelab Sq., 1417614411, Tehran, Iran.
Tel: +98 21 64122111, Fax: +98 21 66461178. E-mail: khmabdi@tums.ac.ir

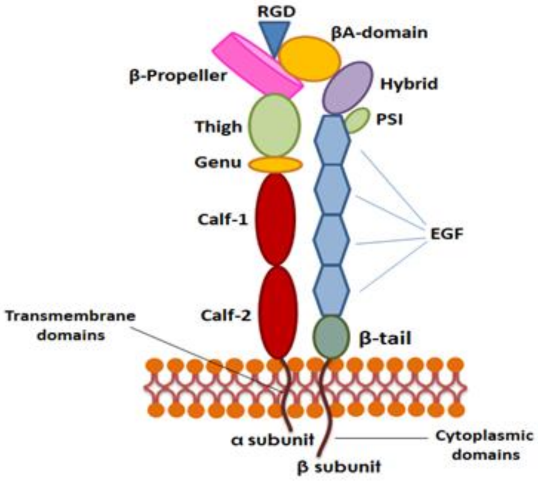

Figure S1. Schematic representation for the extracellular part of integrin $\alpha_v\beta_3$. RGD ligands binding site is the interface between βA-domain of the β_3 subunit and β-propeller of α_v-subunit.

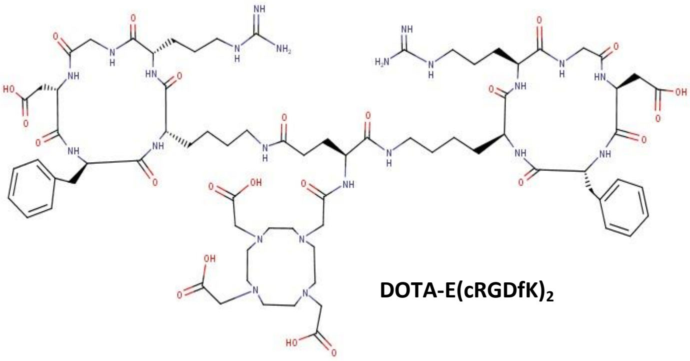

Figure S2. The chemical structure of DOTA-E(cRGDfK)$_2$ based on amino acid sequence.

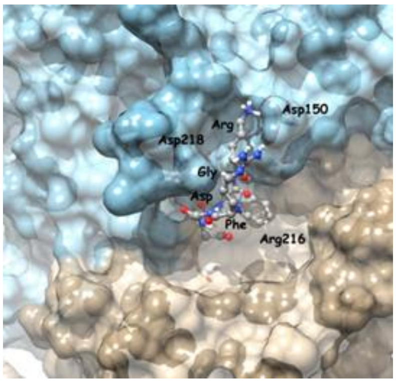

Figure S3. Model for the interaction of cRGDfK with RGD-binding site of $\alpha_v\beta_3$.

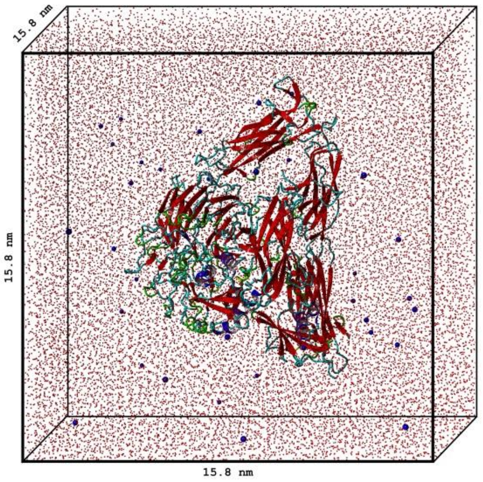

Figure S4. Structure of integrin αvβ3–RGD complex in the simulation 15*15*15 nm box. Red dots and blue spheres represent water molecules and ions, respectively.

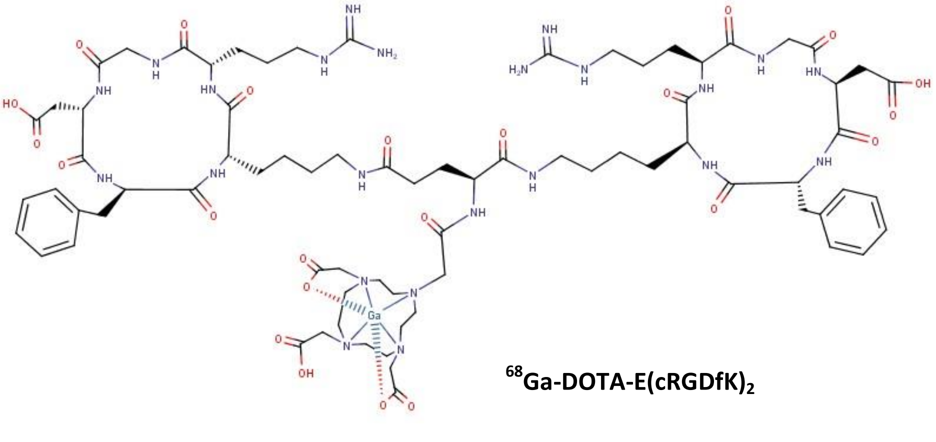

Figure S5. Schematic structure of ^{68}Ga-DOTA-E(cRGDfK)$_2$.

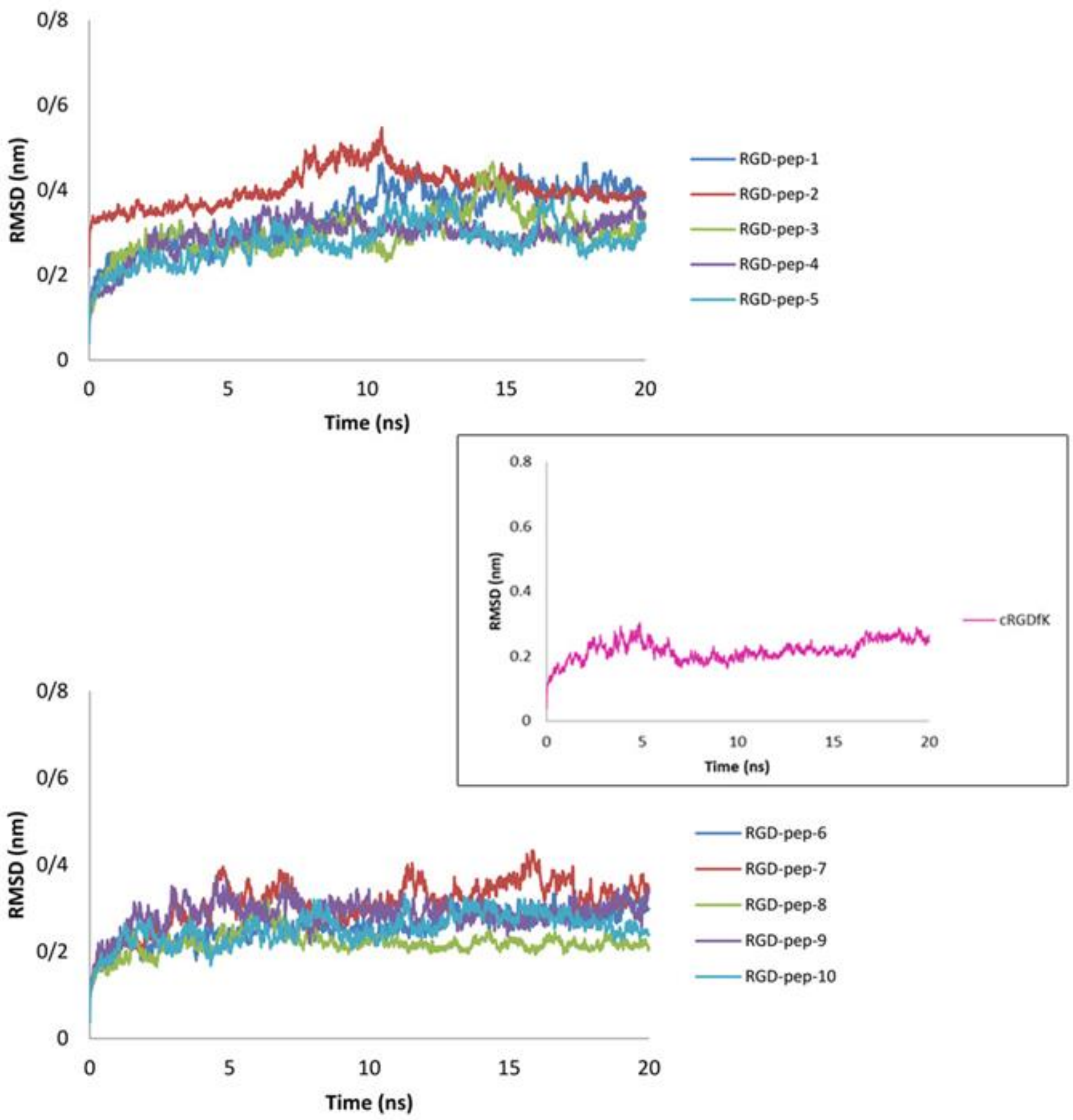

Figure S6. RMSD values (Cα-Cα) during the MD production phase relative to the starting structure during 20 ns total simulation time.

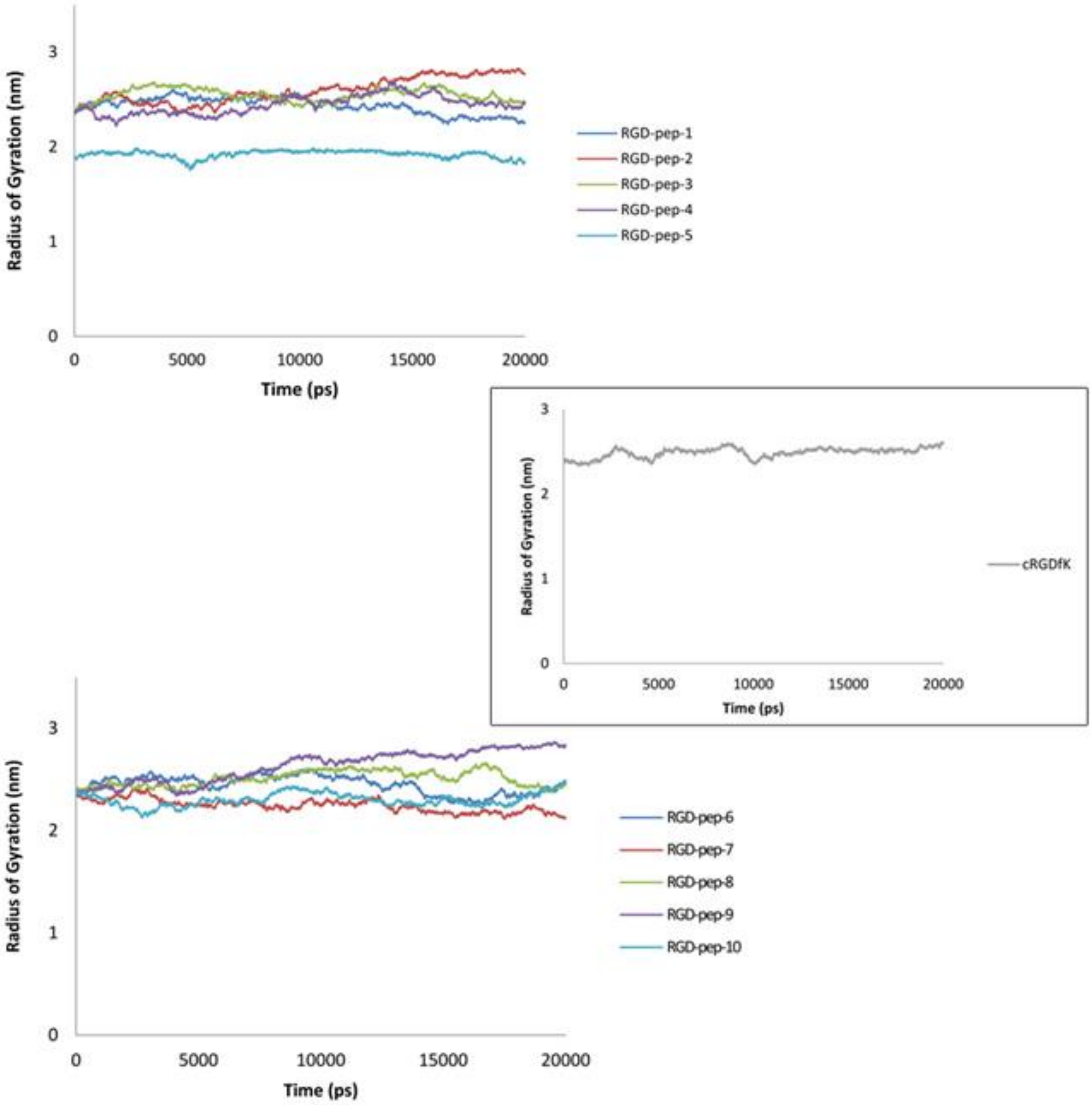

Figure S7. Radius of Gyration as a function of time with respect to the starting structures during MD simulations.

Table S1. Selected sequences for docking, number of amino acids and HADDOCK score.

Name	Sequence	HADDOCK score
Pep-1	GPHGPHGFHGERGPHGPHGPH	-130.5 +/- 20.4
Pep-2	ACDCRGDCFCG	-144.2 +/- 11.5
Pep-3	CRVVRGDTLDC	-137.7 +/- 2.0
Pep-4	CRGDSC	-128.1 +/- 6.2
**Pep-5**	**AVRGDGPKRE**	**-156.4 +/- 7.0**
**Pep-6**	**CRIARGDWNDDRC**	**-148.7 +/- 6.9**
**Pep-7**	**CRPRGDNGDTAC**	**-147.7 +/- 8.0**
**Pep-8**	**AGGLAIAVEGPSKAEISFED**	**-151.0 +/- 6.6**
Pep-9	VMRVDEKTKEVIQ	-141.9 +/- 8.0
**Pep-10**	**IARGDWND**	**-158.8 +/- 4.7**
Pep-11	AWRSDEALPLGS	-145.5 +/- 7.4
Pep-12	HHLGGAKQAGDV	-144.6 +/- 3.1
Pep-13	RGDN	-138.5 +/- 6.3
Pep-14	GP(Hyp)GP(Hyp)GF(Hyp)GERGP(Hyp)GP(Hyp)	-139.3 +/- 6.0
Pep-15	GRGDSPAS	-125.8 +/- 1.7
**Pep-16**	**VIARGDWN**	**-158.4 +/- 3.0**
**Pep-17**	**VTPRGDWNEG**	**-155.5 +/- 3.3**
**Pep-18**	**GRGDLGRLKK**	**-152.8 +/- 5.7**
Pep-19	AGDV	-120.0 +/- 7.6
Pep-20	RGDINNV	-121.7 +/- 6.7
Pep-21	GRGDG	-141.5 +/- 4.1
Pep-22	GRGDSP	-129.5 +/- 5.0
Pep-23	CDCRGDCFC	-131.8 +/- 7.2
Pep-24	AAIALRGDLALLA	-141.8 +/- 9.0
Pep-25	AIALRGDLALL	-128.4 +/- 2.8
Pep-26	IALRGDLAL	-137.6 +/- 4.6
Pep-27	AAIALRGDLALL	-135.4 +/- 10.7
**Pep-28**	**GRRGDLATIHG**	**-161.7 +/- 6.3**
**Pep-29**	**CRIAKGDWNDDRC**	**-157.9 +/- 5.4**
Pep-30	RGDWPC	-143.1 +/- 2.5
**cRGDfK**	**cRGDfK**	**-137.1 +/- 1.8**
E(cRGDfK)2	E(cRGDfK)2	~ 2 × (-137.1 +/- 1.8)

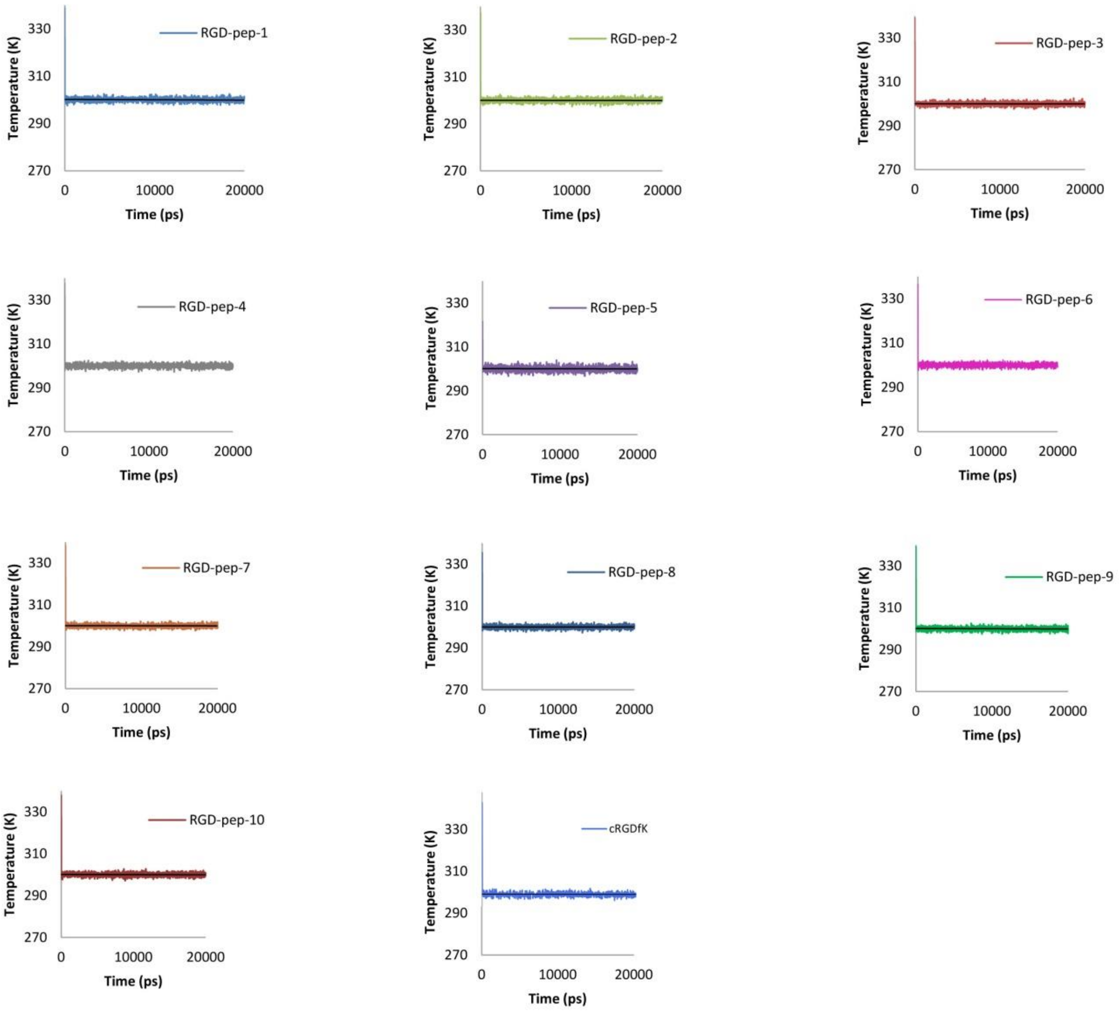

Figure S8. Plots of the system temperature during simulations of the sequences.

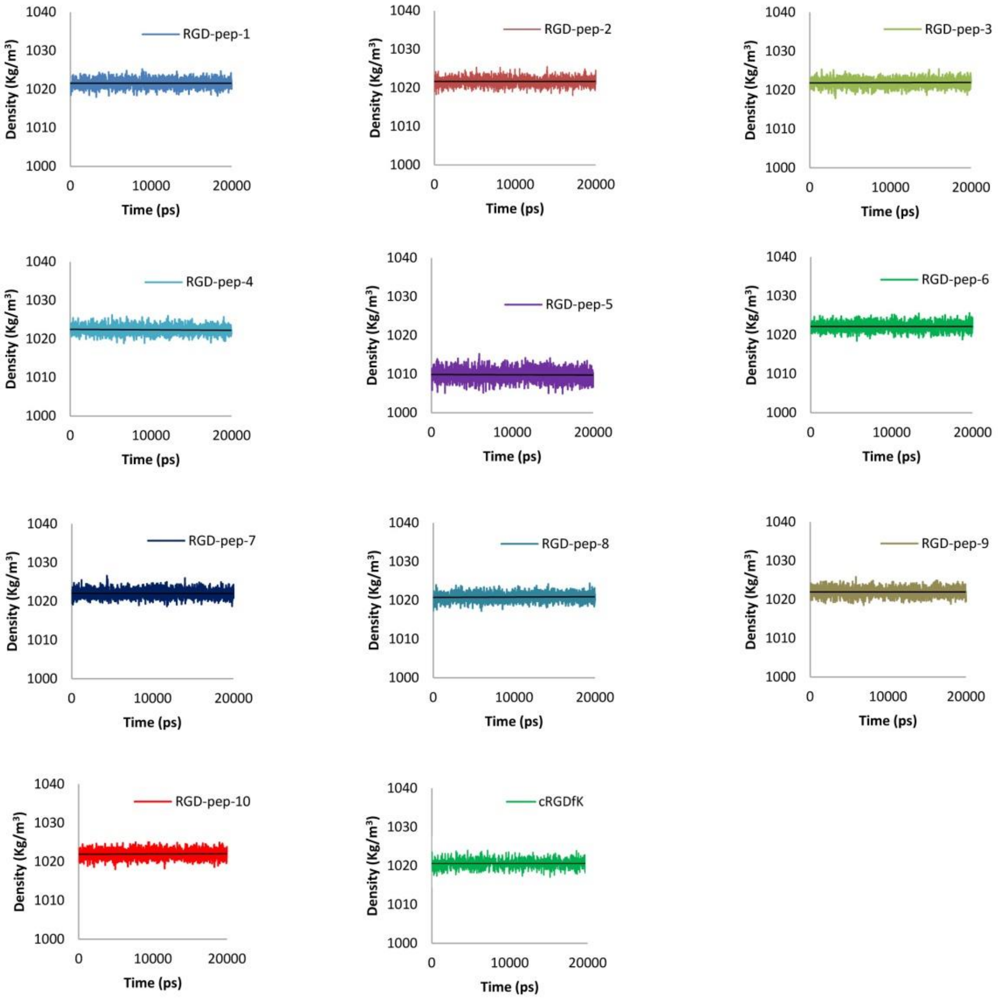

Figure S9. Plots of the system density during MD simulations.

Table S2. Amino acid sequences, number of amino acids and average total energy of the selected sequences.

Name	Amino acid sequence	Number of amino acids	Total energy	Average total RMSF
**E(cRGDfK)2**	**E(cRGDfK)2**	**11**	**-2308.72**	**0.37**
**pep-18**	GRGDLGRLKK	10	-2211.54	0.137
**pep-5**	AVRGDGPKRE	10	-1300.94	0.133
**cRGDfK**	cRGDfK	5	-1154.36	0.361
**pep-28**	GRRGDLATIHG	11	-1065.73	0.351
**pep-6**	CRIAKGDWNDDRC	13	-710.14	0.49
**pep-7**	CRPRGDWNDTAC	12	-535.033	0.286
**pep-16**	VIARGDWN	8	-413.85	0.657
**pep-7**	CRPRGDNGDTAC	12	-346.066	0.361
**pep-8**	AGGLAIAVEGPSKAEISFED	20	81.725	0.366
**pep-10**	IARGDWND	8	120.998	0.436
**pep-17**	VTPRGDWNEG	10	250.985	0.416
